# Effect of radiofrequency radiation from Wi-Fi devices on mercury release from amalgam restorations

**DOI:** 10.1186/s40201-016-0253-z

**Published:** 2016-07-13

**Authors:** Maryam Paknahad, S. M. J. Mortazavi, Shoaleh Shahidi, Ghazal Mortazavi, Masoud Haghani

**Affiliations:** Department of Oral and Maxillofacial Radiology, School of Dentistry, Shiraz University of Medical Sciences, Shiraz, Iran; Medical Physics and Medical Engineering Department, School of Medicine, Shiraz University of Medical Sciences, Imam Hossein Square, 7134845794 Shiraz, Iran; Ionizing and Non-ionizing Radiation Protection Research Center (INIRPRC), Shiraz University of Medical Sciences, Shiraz, Iran; Biomaterial Research Center, School of Dentistry, Shiraz University of Medical Sciences, Shiraz, Iran

**Keywords:** Amalgam, Wi-Fi, Mercury release, Radiofrequency, Electromagnetic fields

## Abstract

**Background:**

Dental amalgam is composed of approximately 50% elemental mercury. Despite concerns over the toxicity of mercury, amalgam is still the most widely used restorative material. Wi-Fi is a rapidly using local area wireless computer networking technology. To the best of our knowledge, this is the first study that evaluates the effect of exposure to Wi-Fi signals on mercury release from amalgam restorations.

**Methods:**

Standard class V cavities were prepared on the buccal surfaces of 20 non-carious extracted human premolars. The teeth were randomly divided into 2 groups (*n* = 10). The control group was stored in non-environment. The specimens in the experimental groups were exposed to a radiofrequency radiation emitted from standard Wi Fi devices at 2.4 GHz for 20 min. The distance between the Wi-Fi router and samples was 30 cm and the router was exchanging data with a laptop computer that was placed 20 m away from the router.

The concentration of mercury in the artificial saliva in the groups was evaluated by using a cold-vapor atomic absorption Mercury Analyzer System. The independent *t* test was used to evaluate any significant differences in mercury release between the two groups.

**Results:**

The mean (±SD) concentration of mercury in the artificial saliva of the Wi-Fi exposed teeth samples was 0.056 ± .025 mg/L, while it was only 0.026 ± .008 mg/L in the non-exposed control samples. This difference was statistically significant (*P* =0.009).

**Conclusion:**

Exposure of patients with amalgam restorations to radiofrequency radiation emitted from conventional Wi-Fi devices can increase mercury release from amalgam restorations.

## Background

Dental amalgam is still the most widely used restorative material in the last 150 years especially in posterior teeth because of its high mechanical strength, durability, ease of manipulation, and low cost [1–5]. Dental amalgam is an alloy comprised of 50 % elemental mercury and a mixture of other metals such as silver, tin, copper, and sometimes palladium, indium and zinc [6–8]. Dental amalgam is considered as the primary source of continuous mercury exposure in general population [1, 9–11]. Mercury is a toxic element which can damage various organs such as central nervous system, renal, respiratory and hematologic systems [12, 13]. Because of the mercury toxicity, the use of mercury has been banned in some European countries [14]. The amount of mercury which releases from amalgam restorations depends on several factors such as number and size of the fillings, composition of amalgam, any other factors that causes load over the restorations like tooth brushing, chewing habits,and bruxism [8, 15].

Wi-Fi is a local area wireless computer networking technology and has been used drastically in houses and public places such as schools and hospitals during recent years [16]. It allows electronic devices such as personal computers, video-game consoles, smart phones, digital cameras and tablet computers to network using Institute of Electrical and Electronics Engineers (IEEE) 802.11 standards. These standards mainly use the 2.5 gigahertz (12 cm) UHF and 5 gigahertz (6 cm) SHF ISM radio bands [17]. The lower cost and easier deployment of these devices than wired computer networks lead to rapidly increase of Wi-Fi devices [18]. However, this also raised great public concern about the potential adverse effects of exposure to electromagnetic fields (EMFs) emitted from these devices [19].

The adverse health impacts associated to exposure to some common sources of electromagnetic fields including laptop computers, mobile phones, MRI and mobile phone jammers have been evaluated by our laboratory in our previous investigations [20–24]. To the best of our knowledge, this is the first study that evaluates the effect of exposure to Wi-Fi signals on mercury release from amalgam restorations.

## Methods

### Teeth samples

This study was approved by the Ethics Committee of Shiraz University of Medical Sciences. Twenty non-carious premolar teeth which were extracted as a part of orthodontic treatment were used in this study. The teeth were stored in isotonic saline solution for not longer than 3 months after surface debridement. The teeth were randomly divided into 2 groups of exposure and control, each containing 10 teeth.

### Amalgam fillings

Standard class V cavities (3mm length, 2mm depth and 5 mm width) were prepared on the buccal surface using carbide burs (SS White Burs, Lakewood, NJ) and a high speed turbine under water spray. The cavities were restored with Cinalux (non-gama-2, spherical amalgam, Faghihi Dental, Tehran, Iran) amalgam. The amalgams were triturated according to manufacturers’ directions, and then they were condensed incrementally towards the cavity walls. All the procedures for restoration of the cavities were performed by the same clinician. The restored teeth were plunged in saline solution at 37° C for 14 days because as it was discussed by Muller Miny et al., the mercury release from amalgam restorations decrease gradually to a constant level 14 days after the filling [25]. Following that and before exposing the teeth, samples were poured into plastic tubes filled with artificial saliva. The thickness of the artificial saliva covered over teeth samples was 1.5 cm to mimic soft tissue.

### Wi-Fi exposure

**Fig. 1:**
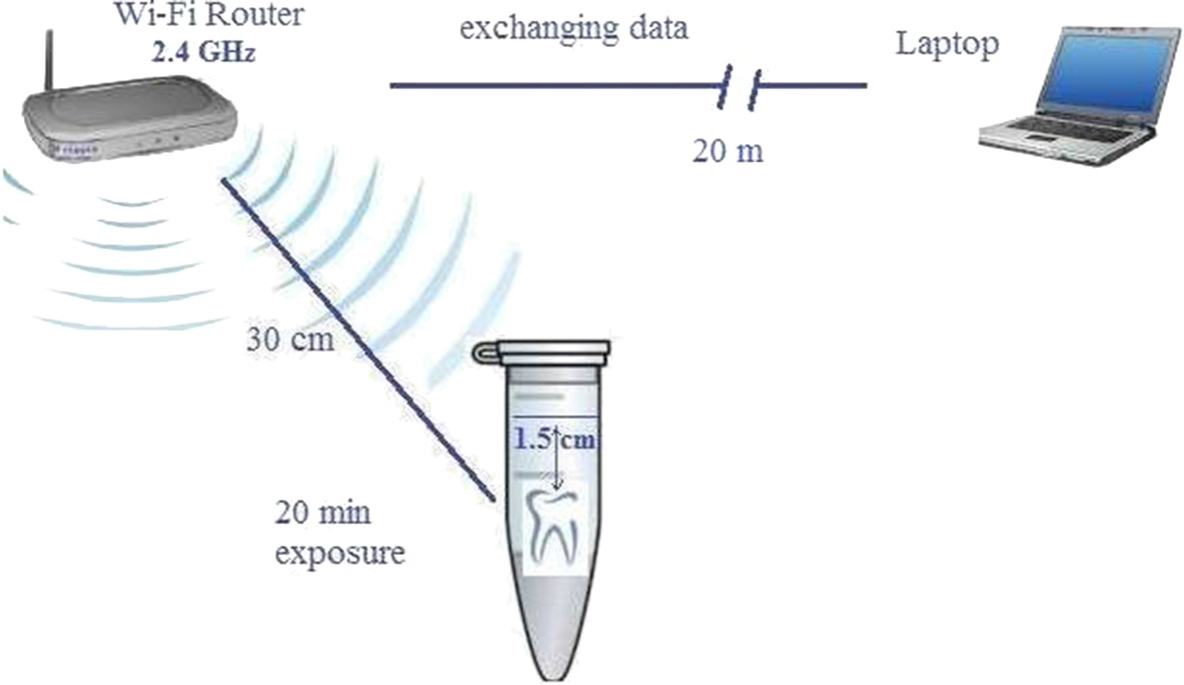
The geometry used for exposure of the teeth samples

The exposure group was exposed to radiofrequency radiation emitted from standard Wi-Fi devices at 2.4 GHz for 20 min. The distance between the Wi-Fi router (D-Link, China) and samples was 30 cm and the router was exchanging data with a laptop computer that was placed 20 m away from the router. The control group was kept outside the experiment room. The geometry used for exposure is shown in Fig. 1.

### Mercury measurement

Based on our previous experiments, it was clearly revealed that the pre-exposure mercury concentration in the saliva containing teeth samples with exactly identical fillings (the same cavities and amalgam type), was the same for all samples (the differences were not statistically significant). Therfore, the mercury levels were measured in the artificial saliva after exposure by cold vapor atomic absorption spectrometry (CVAAS; Analytical Jena, vario 6, Germany).

### Statistical analysis

The data were statistically analyzed using SPSS version 16.0 (SPSS Inc., Chicago, IL) (http://www-01.ibm.com/software/analytics/ spss). The independent *t* test was used to compare the level of mercury release in the exposure and control groups to identify any statistically significant differences. *P* value <0.05 was considered significant.

## Results

Descriptive statistics were presented as the mean, standard deviation, minimum and maximum in Table 1. The mean (±SD) concentration of mercury in the artificial saliva of the Wi-Fi exposed group was 0.056 ± .025 mg/L, while it was only 0.026 ± .008 mg/L in the non-exposed control samples. Therefore, the mean concentration of mercury in the Wi-Fi group was about twice of the control group. The observed difference in the concentration of mercury in the artificial saliva of the exposure and control group was statistically significant (*P* =0.009).

**Table 1:** The mean, standard deviation, minimum and maximum of the mercury release in the two groups

Mercury release (mg/L)	Group		(*P*-value)
	Control	Wi-Fi	
Mean ± SD (Range: min -max)	0.026 ± .008 (0.016 – 0.039)	0.056 ± .025 (0.020-0.100)	0.009

## Discussion

Public concern about the possible adverse health effects of using Wi-Fi technology is increasing because of the widespread use of wireless communication systems [19]. In the present study, it was concluded that radiofrequency radiation emitted from Wi-Fi devices significantly increased mercury release from amalgam restorations.

Mortazavi and Mortazavi have recently reviewed the published reports on the increased release of mercury from dental amalgam fillings after exposure to different sources of electromagnetic fields (e.g. MRI, mobile phones) [26]. These studies are summarized in Table 2. The first report on the role of exposure to MRI or microwave radiation emitted by mobile phones in increasing the release of mercury from dental amalgam filling was published by Mortazavi et al. in 2008 [27]. To overcome the limitations of their previous study, Mortazavi and his colleagues have recently studied the effects of stronger magnetic fields (1.5 T in their recent study vs. 0.25 T in their previous report). This study confirmed the previous findings and provided further support for increased release of mercury from dental amalgam fillings after MR imaging [28].

**Table 2:** Comparison of the findings of current study with other studies performed either on mercury release or amalgam microleakage after exposure to electromagnetic fields

Radiation source	Endpoint	Methods	Basic finding	Reference
Mobile Phone	Release of Mercury	Urine samples were collected from 14 female students	A statistically significant (*p* < 0.05) higher concentration of mercury was observed in the students who used mobile phones.	Mortazavi et al. [27]
MRI (0.23 T)	Release of Mercury	Stimulated saliva collected in 30 persons	Elevated urinary mercury concentration in the exposed group	Mortazavi et al. [27]
MRI (1.5 T)	Release of Mercury	Urinary concentrations of mercury in the MRI exposed and control subjects	The urinary mercury in the exposed group, 72 h after MRI (96 h after restoration),was significantly higher (*p* = 0.046).	Mortazavi et al. [28]
X-ray	Release of Mercury	Teeth samples were exposed to X-rays in a soft tissue-equivalent material	A significant increase in mercury was observed in the X-ray-exposed group (*p* ≤ 0.05).	Kursun et al. [42]
MRI	Release of Mercury	Teeth samples were exposed to MRI in a soft tissue-equivalent material	No significant difference was found in the MRI-exposed group.	Kursun et al. [42]
MRI (3 T)	Microleakage of amalgam	60 extracted teeth divided into experimental and control groups exposed/shamexposed to a magnetic field of 3 T for 20 min	significant differences in microleakage between the groups exposed to MRI and controls, whereas differences in microleakage between amalgam types were insignificant.	Yilmaz and Misirlioglu [30]
MRI (1.5 T)	Microleakage of amalgam	63 human freshly extracted premolars were divided into 3 groups (3 different amalgams). In each group, 50% of the samples were exposed to MRI.	Differences in microleakage within each group following MRI were significant in the GS-80 and Vivacap groups but not in the Cinalux group.	Shahidi et al. [29]
MRI (1.5 T)	Microleakage of amalgam	40 teeth were randomly divided into four groups.. The first and third groups were exposed to MRI.	No significant differences of occlusal and gingival surface microleakage after MRI exposure were observed.	Akgun et al. 2014 [29]
Wi-Fi	Mercury release	20 extracted teeth were randomly divided into 2 groups of Wi-Fi exposure and control.	A significant increase in mercury release was observed in Wi-Fi exposed group.	Current study

It should be noted that the results obtained in the studies performed on the role of exposure to electromagnetic fields in magnetic resonance imaging on the microleakage of amalgam are strongly in line with the findings of Mortazavi et al. [29, 30]. To the best of our knowledge, our current study is the first study that investigates the effect of radiofrequency radiation emitted by Wi-Fi routers on mercury release from amalgam restorations.

Mercury is a toxic element which has adverse biological effects even at low doses [31]. Therefore, it seems to be necessary to apply a sensitive and reliable analytical technique to determine mercury content. Various analytical techniques has been used previously for the determination of mercury in environmental and biological samples such as cold vapor atomic absorption spectrometry (CVAAS), cold vapor fluorescence spectrometry (CVAFS), inductively coupled plasma optical emission spectrometry (ICP OES), electrothermal atomic absorption spectrometry (ET AAS), neutron activation analysis, mass spectrometry, anodic stripping voltammetry, and cold vapor inductively coupled plasma mass spectrometry (CV ICP-MS) [32–35]. This study employed CVAAS method for measuring mercury released from dental amalgam. Because CVAAS is the most widely technique used in previous studies for detecting this element at low concentrations due to its high sensitivity and selectivity and because of its low cost [36, 37].

To improve the outcome of the west possible mercury release, we did not polish the cavities after restoration, because according to Ferracane et al. greater amounts of mercury would release from unpolished than polished surfaces [38].

Although the adverse health effects of the exposure to radiofrequency radiation emitted by Wi-Fi routers on some challenging phenomena such as human reproductive capabilities is well documented by some researchers around the world [39, 40], as far as we know, there is no report on the role of Wi-Fi radiation on the release of mercury from amalgam restorations. The mercury release from dental amalgam into saliva has been evaluated in previous studies both *in vitro* and *in vivo* conditions [25, 31, 41–43]. One of the limitation of *in vivo* studies, as Mortazavi et al. discussed in their study, was that the participants were referred by their own physicians and the investigators did not have control over the number and surface of amalgam fillings [41]. However, in our *in vitro* study, we could control these factors by using identical class V fillings with the same dimensions through application of a template during cavity preparations since the mercury exposure correlates significantly to the number and surface of fillings [8, 15]. We also could control some other confounding factors which differ inter individually such as chewing habits and thermal effects [15, 44]. On the other hand some factors that may decrease the mercury release such as the liberation of corrosive products by contact of food and bacteria did not also interference with our findings.

## Conclusion

To the best of our knowledge, this is the first study which assesses the effect of exposure to Wi-Fi signals on mercury release from amalgam restorations. We speculated that exposure to radiofrequency emitted from Wi-Fi devices may result in mercury release from amalgam restorations. Further *in vitro* and *in vivo* studies are necessary to prove this contention.

## Abbreviations

CV ICP-MS, cold vapor inductively coupled plasma mass spectrometry; CVAAS, cold vapor atomic absorption spectrometry; CVAFS, cold vapor fluorescence spectrometry; EMF, electromagnetic fields; ET AAS, electrothermal atomic absorption spectrometry; ICP OES, inductively coupled plasma optical emission spectrometry; MRI, magnetic resonance imaging; SHF, super high frequency; UHF, ultra high frequency; Wi-Fi, wireless fidelity.
